# The NMR structure of the murine DLC2 SAM domain reveals a variant fold that is similar to a four-helix bundle

**DOI:** 10.1186/1472-6807-7-34

**Published:** 2007-05-22

**Authors:** Jamie J Kwan, Logan W Donaldson

**Affiliations:** 1 Department of Biology, York University, 4700 Keele Street, Toronto, Ontario M3J 1P3, Canada

## Abstract

**Background:**

The tumor suppressor DLC2 (Deleted in Liver Cancer -2) participates in cell signaling at the mitochondrial membrane. DLC2 is characterized by a SAM (sterile alpha motif) domain, a Rho GTPase activating protein (GAP) domain, and a START lipid transfer domain.

**Results:**

Towards understanding the function of DLC2, we have solved the NMR solution structure of the SAM domain. The DLC2-SAM domain structure reveals an atypical four-helix composition that is distinct from the five-helix SAM domain structures that have been determined to date. From structural alignments, helix 3 of the canonical SAM domain appears to be replaced by shorter, extended secondary structure that follows a similar path. Another difference is demonstrated by helices 1 and 2 that form a helical hairpin that is situated approximately parallel to the canonical helix 5.

**Conclusion:**

The DLC2-SAM domain adopts a structure that is topologically more similar to an anti-parallel four-helix bundle than a canonical SAM domain. This alternate topology may allow the DLC2-SAM domain to interact with a novel set of ligands.

## Background

Many tumors demonstrate a characteristic, non-random deletion of chromosomal material, termed loss of heterozygosity (LOH). From molecular genetics studies, *Deleted in liver cancer-1 *(*DLC1*) [1] and a closely related gene, *DLC2 *[2] were discovered at two distinct chromosomal loci known to be sensitive to LOH. The DLC1 and DLC2 proteins share 51 % amino acid identity. Several studies suggest that DLC1 and DLC2 are tumor suppressors involved in the progression of a wide range of cancers [3]. For example, reintroduction of DLC1 suppresses proliferation of breast carcinoma [4] and hepatoma cells [5] and prevents the formation of tumors in nude mice. Expression of DLC2 prevents the formation of Ras induced foci in NIH3T3 cells [2]. From examinations of several cancers, point mutations leading to the inactivation of DLC1 are rare. Rather, DLC1 appears to be downregulated by promoter methylation [6].

At 1113 residues, murine DLC2 is a large protein with relatively few identifiable domains. Based upon sequence homology and a recent deletion study [2], the amino terminus of DLC2 contains a Sterile Alpha Motif (SAM) domain [7]. Following an expanse of unknown function, a GTPase activating domain (GAP) capable of inactivating Rho and Cdc42 is situated near the carboxyl terminus [2]. A steroidogenic acute regulatory protein (StAR)-related lipid transfer (START) domain completes the protein and localizes DLC2 to mitochondria that are proximal to lipid droplets [8]. All of these domains are likely to be regulated by an array of intra- and intermolecular protein partnerships. The SAM domain, which has a rich history as a protein-ligand binding motif [9], represents an excellent focal point for exploring DLC2 partnerships in detail.

Towards determining its functional role, we present the structure of the murine DLC2-SAM domain solved using nuclear magnetic resonance (NMR) methods. Consistent with secondary structure predictions, the DLC2-SAM domain lacks what would be the third helix of a canonical, five helix SAM domain. Furthermore, the first two helices occur in a unique orientation. Combined, these differences result in a structure that resembles an anti-parallel four-helix bundle as much as it resembles a SAM domain. Upon closer inspection of the structure, a hydrophobic cleft lined with aromatic residues may offer a binding site for a unique class of ligands.

## Results

**Figure 1:**
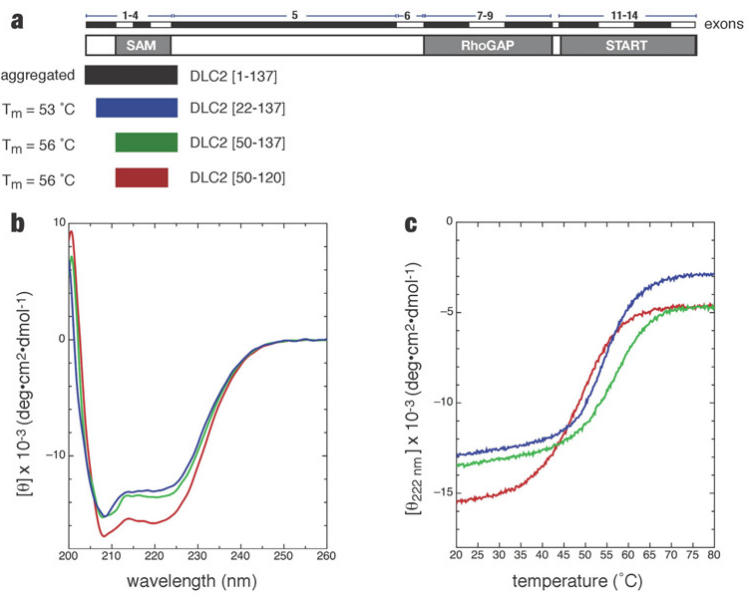
Delineation of the minimal DLC2-SAM domain. *(a) *By sequence homology, the DLC2-SAM domain is defined by exons 2–4. Of four protein fragments expressed, three were suitable for further biophysical analyses. *(b) *Far UV CD spectra. *(c) *Thermal stability was determined by monitoring ellipticity at 220 nm, a wavelength characteristic of α-helical secondary structure.

By sequence similarity, the murine DLC2-SAM domain is located near the N-terminus of the protein with boundaries conspicuously defined by exons 2–4 (aa. 58–129). The first exon has no obvious similarity to any short protein domains (Figure 1a). Using sequence similarity and exon boundaries as a guide, we expressed four hexahistidine-tagged protein fragments spanning the DLC2-SAM domain. Highly purified preparations of the largest fragment, DLC2 (1–137), were aggregated at μM concentrations precluding further analysis. Fortunately, the remaining three DLC2 protein fragments were suitable for biophysical characterization.

Far UV CD spectra indicate that DLC2 (22–137), DLC2 (50–120) and DLC2 (50–137) were all folded with a majority of α-helical content as shown by two characteristic minima at 208 and 222 nm (Figure 1b). In exon 1 (aa. 1–57), the PHD secondary structure algorithm [10] predicts the presence of one α-helix spanning residues 22–36 immediately preceding the start of the SAM domain. Assuming an α-helix is present in exon 1, the predicted mean residue ellipticities (MRE) at 222 nm for DLC2 (50–120), DLC2 (22–137) and DLC2 (50–137), are 15100, 12700, and 13700 deg·cm^2^·dmol^-1^, respectively. Alternatively, if no α-helix is present in exon 1 of DLC2 (22–137), the predicted ellipticity decreases to 10100. Together, the three spectra presented in Figure 1a appear to underestimate the predicted MREs by ~10%, which may be due in part to a systematic error in the estimation of protein concentration. Regardless, at a qualitative level, the similarity in the absolute MREs between DLC2 (22–137) and DLC2 (50–137) suggest that an α-helix may be present in exon 1.

The shortest fragment, DLC2 (50–120) demonstrated a thermal denaturation midpoint (or melting point) that was approximately 5°C lower than DLC2 (22–137) and DLC2 (50–137) suggesting that residues beyond Arg120 contribute stabilizing contacts. Since DLC2 (22–137) and DLC2 (50–137) demonstrated similar melting points, it suggests that the N-terminal sequence immediately flanking the DLC2-SAM domain does not provide any additional stabilizing contacts.

Following the CD study, the three DLC2-SAM protein fragments were ^15^N-labeled for NMR analysis. Amide HSQC spectra of DLC2 (22–137) and DLC2 (50–137) were nearly identical both in terms of the number of amide resonances and their absolute chemical shifts (data not presented). The similarity between the two spectra suggests that residues 22–49 occur in intermediate exchange and therefore do not interact with the SAM domain. A comparison of the DLC2 (50–137) and DLC2 (50–120) HSQC spectra revealed several sharp resonances that were not present in DLC2 (50–120). In the absence of chemical shift assignments, these sharp resonances would be attributed to a disordered stretch extending from the C-terminal end of the SAM domain. Taken together, these CD spectra and NMR HSQC spectra indicate that the minimal protein fragment of DLC2 encompassing the SAM domain is consistent with the boundaries (aa. 58–129) predicted by exons and sequence similarity.

While the spectra of DLC2 (50–120) were qualitatively easier to interpret due to the absence of sharp resonances and the reduced ^1^H chemical shift degeneracy, this protein fragment could only be concentrated to 0.3 mM thereby precluding any structural studies. Furthermore, preparations of DLC2 (50–120) precipitated when mixed with Pf1 bacteriophage for NMR orientational studies. In contrast, preparations of DLC2 (50–137) were stable up to a concentration of 1 mM and could be mixed with Pf1 bacteriophage. Based upon greater solubility and thermal stability, DLC2 (50–137) was selected for structural studies.

The NMR structure of the murine DLC2-SAM domain (50–137) was solved using a combination of experimentally derived NOE distance restraints, amide residual dipolar couplings, and dihedral angles derived from chemical shifts (Table 1). The NMR structure of DLC2 (50–137) indicates that the last ordered residue is Ala117. In contrast, the CD study suggested there were additional stabilizing determinants beyond Arg120. However, we did not observe any contributions to the hydrophobic core from the nearby hydrophobes Leu121, Val123, or Phe125. The ensemble of lowest energy structures spanning residues 58–117 has a global backbone atom precision of 1.10 Å. Restricted to the regular secondary structure, the backbone atom precision is 0.64 Å (Figure 1a).

**Table 1:** Statistics for the ensemble of structures of the murine DLC2 SAM domain^*a*^

Distance restraints	
Intraresidue	377
Sequential (|i-j| = 1)	172
Medium range (2 ≤ |i-j| ≤ 4	76
Long range (4 < |i-j|)	95
Hydrogen bond pairs (HN-O, N-O)	42
Residual Dipolar Couplings	
H-NH	23
NOE violations	
> 0.5 Å	0.0 ± 0.0
> 0.3 Å	18.0 ± 3.2
Dihedral angle restraints^*b*^	
φ/ψ angles for each amino acid	57
Deviations from standard geometry (XPLOR-NIH)	
Bonds	0.0125 ± 0.0004
Angles	1.4838 ± 0.0399
Impropers	1.9898 ± 0.2513
Pairwise RMSD (Secondary structure^*c*^)	
Backbone	0.64 ± 0.11 Å
All heavy atoms	1.47 ± 0.16 Å
Ramachandran Statistics^*d*^	
Most favored regions	86.6 %
Additional allowed regions	12.0 %
Generously allowed regions	1.4 %
Disallowed regions	0.0 %

^*a*^Ensemble of the top 20 structures with lowest overall energy and number of restraint violations.

^*b*^Predicted from chemical shifts using the PREDITOR web server.

^*c*^RMSD values for residues 58–69, 73–81, 88–94, 103–117.

^*d*^Determined with PROCHECK-NMR for 10 lowest-energy structures.

**Figure 2:**
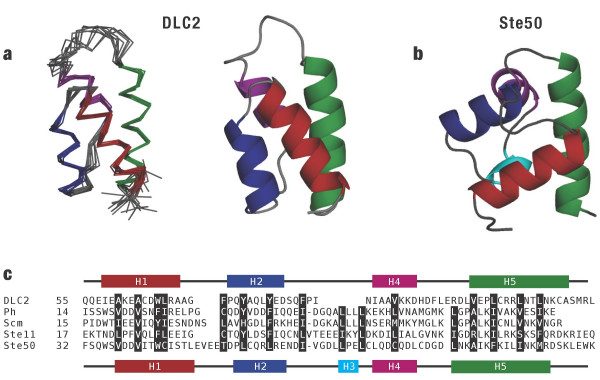
A comparison of DLC2-SAM domain and other SAM domains. *(a) *A representative structure of the DLC2-SAM domain in ribbon form and as ensemble of the 10 lowest energy solutions. A short helix H3 (cyan) typically observed in SAM domains such as *(b) S. cerevisiae *Ste50 [PDB: 1Z1V] is absent in the DLC2-SAM domain. *(c) *Sequence alignment of murine DLC2 with SAM domains known to participate in protein-protein interactions. Listed below DLC2 are SAM domains from *Drosophila *Polyhomeotic (Ph), *Drosophila *Sex-Comb-on-Midleg (Scm), *S. cerevisiae *Ste11 and Ste50. Hydrophobic amino acids that contribute to the hydrophobic core are highlighted.

Previously, we solved the NMR structure of the *S. cerevisiae *Ste50-SAM domain [11]. Based upon sequence similarity, number of helices and the position of the helices, the Ste50-SAM domain demonstrates a typical fold and therefore, is a good basis for comparison with the DLC2-SAM domain structure. As well, Ste50, like other SAM domains including *Drosophila *Ph, Scm, Yan and Mae [9] presents two complementary surfaces for high-affinity SAM-SAM interactions. Overall, the SAM domains of DLC2 and Ste50 [PDB: 1Z1V] superimposed with a Cα root-mean-square deviation (RMSD) of 3.1 Å over 42 residues (Z-score = 2.5). As illustrated in Figure 2b, the DLC2-SAM domain differs from a canonical, five-helix SAM domain fold of Ste50 in two distinct ways.

The first immediate distinction between the DLC2 and Ste50-SAM domains is demonstrated by the difference in the number of helices present. Helix H3, which is typically short, is replaced in the DLC2-SAM domain by an extended structure occupying the same location. To facilitate the comparison with Ste50, we will refer to the DLC2-SAM domain as a four-helix fold comprised of helices H1, H2, H4 and H5. In the Ste50-SAM domain, Leu59 and Ile 61 anchor helix H3 and contribute to the compact, hydrophobic core. As shown in Figure 2b, two regularly spaced, conserved hydrophobic residues also anchor helix H3 of the *S. cerevisiae *Ste11-SAM domain [12] and the *Drosophila *Polyhomeotic (Ph) and Sex-Comb-on-Midleg (Scm) SAM domains [13]. In lieu of helix H3, Ile84 and Ile88 supply the requisite hydrophobic contacts from nearby positions in the DLC2-SAM domain (Figure 2c).

The relative orientation of a hairpin defined by helices H1 and H2 represents a second major distinction between the Ste50 and DLC2-SAM domains. In a typical SAM domain, the hairpin is approximately perpendicular to helix H5. In contrast, the helical hairpin of DLC2-SAM occurs in an anti-parallel orientation to helix H5. Key unambiguous experimental NOE observations defining this orientation are derived from Tyr69 in helix H2 (Hδ = 6.40 ppm, Hε = 6.65 ppm) and Leu103 in helix H5 (Hδ1 = -0.18 ppm, Hδ2 = 0.30 ppm). Both of these residues are distinguished by their upfield chemical shifts. Interestingly, the side chain of Trp66, a bulky residue conserved throughout all SAM domains in helix H1, is similarly positioned near helix H5 in both DLC2-SAM and Ste50-SAM domain structures. Thus, Trp66 may be considered a pivot point through which the helical hairpin has been rotated.

**Figure 3:**
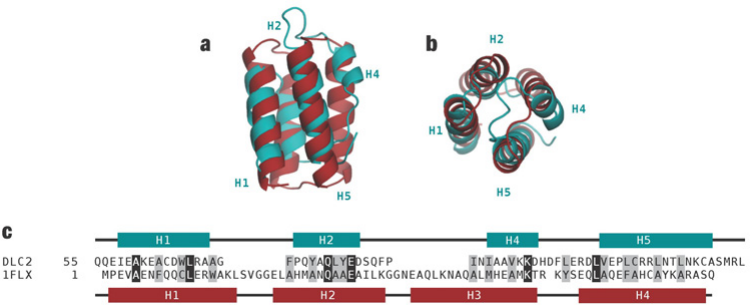
A comparison of the DLC2-SAM domain (in teal) and FELIX (in magenta), a model of a engineered four-helix bundle [PDB: 1FLX]. (*a, b*) Two views of a best-fit superimposition of the DLC2 and FELIX structures. While helix H4 of DLC2 is much shorter than its analogous helix (H3) in FELIX, its observed length is consistent with other SAM domains. *(c) *A sequence alignment highlighting identical (black) and homologous (grey) residues.

Pairwise comparisons between the DLC2-SAM domain and the entire PDB were performed using the SSM (Secondary Structure Matching) server at the European Bioinformatics Institute [14]. The SSM method assigns a quality score to each match that is a function of overall protein length, the number of aligned residues between the two proteins, the number of gaps introduced and the Cα RMSD. From this survey, no SAM domains were identified among the top 100 hits presumably due to the alternate placement of the helices H1/H2 and the absence of helix H3. The top hit with a RMSD of 2.40 Å over 51 aligned residues was FELIX [PDB: 1FLX], a theoretical model of an anti-parallel, or up-down-up-down, four-helix bundle [15]. A superimposition of FELIX and DLC2-SAM demonstrating the structural similarity is shown in Figures 3a and 3b. Although only six residues are identical between FELIX and DLC2-SAM, the amphipathic character of the four helices is retained and consequently, the positions of nonpolar residues that contribute to the hydrophobic core of the respective proteins (Figure 3c). On the basis of RMSD (2.53 Å) and most aligned residues (53), the best experimentally determined structure that was similar to the DLC2-SAM domain is also a four-helix bundle termed S-824 (PDB: 1P68) [16].

Interhelical angles present a straightforward means of comparing the DLC2-SAM domain with representatives of four-helix bundle and five-helix SAM domain classes. As shown in Table 2, the DLC2-SAM domain presents its four helices in a nearly parallel manner, although there is variation in the direction at which the helices cross as compared to the FELIX model and the S-824 protein structure. There appears to be no similarity between the interhelical angles of DLC2-SAM and nine five-helix SAM domains presented. However, within the five-helix SAM domain class itself, there is considerable similarity, the only exception being the angle at which helices H3 and H4 cross each other.

**Table 2:** Interhelical angles of selected four-helix bundles and SAM domains^*a*^

PDB^*b*^	Name	Helix Pair^*c*^					

		H1-H2	H2-H3	H3-H4	H4-H5	H5-H1	H2-H4
		
2JMT	DLC2	-163	NA	NA	156	147	-150
1P68	S-824	177	NA	NA	-156	-174	-159
1FLX	FELIX	-164	NA	NA	-165	-166	-160

1SVO	Mae	138	75	109	141	124	NA
1SV4	Yan	138	95	108	138	124	NA
1F0M	EphB2	153	95	108	138	124	NA
1DXS	p73	154	96	112	129	124	NA
1PK3	Scm	162	102	112	135	75	NA
1KW4	Ph	161	112	-87	155	118	NA
1B0X	EphA4	149	97	-113	127	118	NA
1OW5	Ste11	145	100	-114	112	132	NA
1Z1V	Ste50	158	101	-116	121	115	NA
		
	Average^*d*^	151	97	13	132	117	
	SD^*e*^	9	10	115	12	17	

^*a*^Calculated with the program INTERHLX

^*b*^Protein Data Bank accession number

^*c*^To facilitate a comparison with five-helix SAM domains, the helix order of 2JMT, 1FLX and 1P68 is defined as H1-H2-H4-H5

^*d*^Average angles are calculated only for the five-helix SAM domains

^*e*^Standard deviations are calculated only for the five-helix SAM domains

A dynamics study was performed to supplement the DLC2-SAM domain structure determination. Amide ^15^N T_1 _and T_2 _relaxation times and heteronuclear NOE enhancements were measured at 23°C. In general terms, T_1 _relaxation rates and heteronuclear NOE enhancements tend to be sensitive to fast motions at the ns timescale. On the other hand, T_2 _relaxation rates are sensitive to slower processes. A global molecular tumbling time, or correlation time, can be determined from the ensemble of T_1 _and T_2 _rates. As larger molecules tumble slower, the correlation time provides insight into the oligomeric state of a given protein. From gel filtration studies performed at μM concentrations, the DLC2-SAM domain elutes as a single peak with a retention time that is consistent with a monomeric protein (data not presented).

At 1.3 mM, the concentration used for the NMR structural study, the average T_1_/T_2 _ratio was 10.96 ± 1.35 reflecting a correlation time (t_c_) of 10.3 ns. When the sample was diluted in half to 0.65 mM and then in half again to 0.33 mM, the T_1_/T_2 _ratios dropped slightly to 9.88 ± 1.22 and 9.85 ± 1.26 with respective correlation times of 9.44 ns and 9.46 ns. At a similar protein concentration and ionic strength, the *S. cerevisiae *Ste11 kinase SAM domain demonstrates a comparable correlation time of 9.3 ns [17]. The observed reduction in correlation times suggests that the DLC2-SAM domain has a slight concentration dependent propensity to self-associate. Supporting this conclusion, DLC2-SAM domain preparations for NMR spectroscopy were observed to polymerize into a gel over a period of months, even at 4°C and in the presence of reducing agents to prevent oxidization of the three solvent exposed cysteines. Weak self-association has been documented previously for the Ste11 [17] and human Ephrin kinase B2 SAM domains [18]. However, unlike these two SAM domains, no concentration dependent chemical shift changes were observed suggesting that DLC2-SAM self-association is much weaker. At low concentrations (0.3 mM), the minimal DLC2-SAM protein fragment (50–120) had sufficient solubility for a relaxation study to be performed. From a similar T_1 _and T_2 _relaxation rate analysis, the loss of 17 C-terminal residues reduced the global correlation time from 9.46 to 7.83 ns.

**Figure 4:**
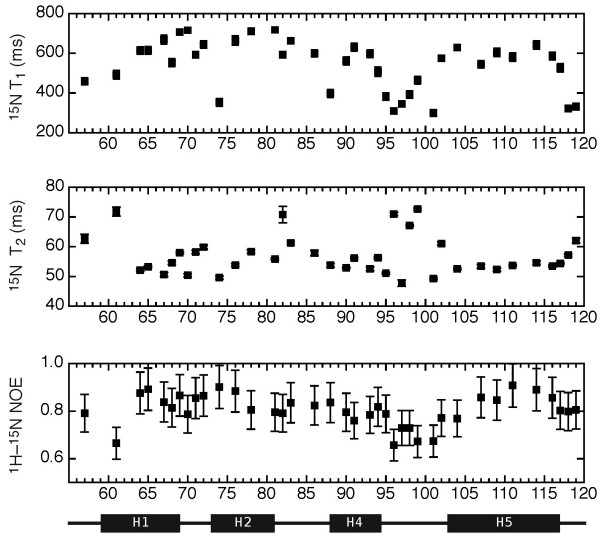
A NMR relaxation study of the DLC2-SAM domain. *(a) *Per residue ^15^N T_1 _longitudinal relaxation times. *(b*) Per residue ^15^N T_2 _transverse relaxation times. *(c) *Per residue heteronuclear NOE ratios.

On a per residue basis, the helix H4-H5 loop demonstrated higher T_1 _and T_2 _relaxation times and lower heteronuclear NOE values (Figure 4) indicating the contribution of additional motions in the μs-ms range. Consistent with the greater relative conformational sampling occurring in this region, fewer NOE observations were made thereby leading to a higher backbone RMSD in the ensemble.

**Figure 5:**
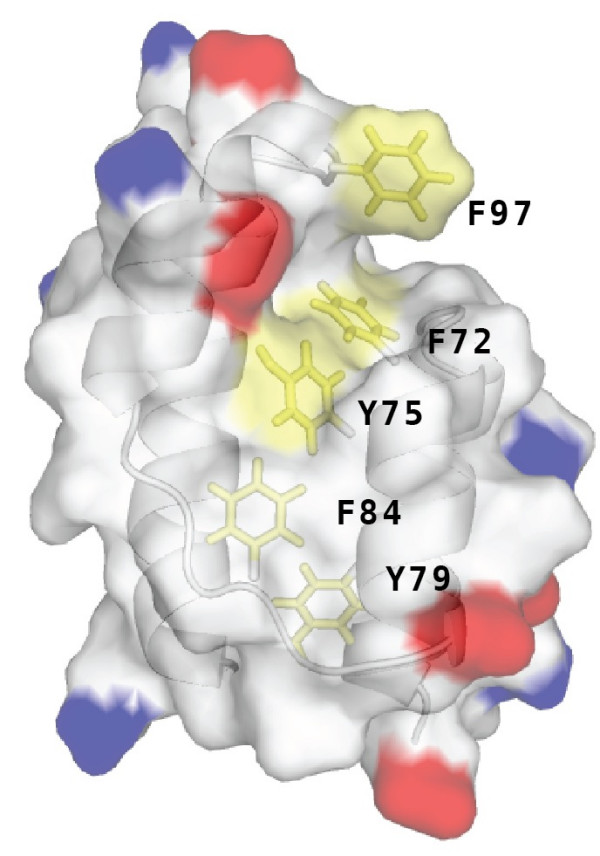
A molecular surface representation of the DLC2 SAM domain was colored according to charge (Asp/Glu, red; Lys/Arg, blue; aromatics, yellow; all others, white). With helices H2 and H4 immediately towards the viewer, a shallow cleft lined with aromatic residues is apparent.

An examination of the DLC2-SAM domain surface reveals a narrow hydrophobic cleft that extends from the helix H2-H4 loop towards helix H5 (Figure 5). This surface follows a tract of aromatic residues that include Phe72, Tyr75, Tyr79, Phe84 and Phe97. Of these aromatics, Phe72 and Phe75 are partially exposed and Phe97 is entirely solvent exposed thereby providing a shallow pocket for a hydrophobic ligand. The position of this potential ligand binding cleft has not been observed in protein-protein [9] and protein-nucleic acid complexes [19] of other SAM domains.

## Discussion

Convergence and divergence of signals through Ras and Rho represent some of the most studied cases in the literature [20]. By targeting Rho, Cdc42, and possibly other GTPases, the GAP activity of DLC2 exerts a repressive effect on cell proliferation and regulates the formation of actin stress fibers and focal adhesions [21]. Combined, these abilities have earned DLC2 the designation as a tumor suppressor. Since RhoGAP signaling proteins such as DLC2 outnumber their potential GTPase targets by over a factor of 2:1, a considerable level of crosstalk is expected [22]. While the GAP domain of DLC2 possesses all of the necessary determinants to interact with Rho, additional sequences are likely necessary to couple DLC2 to multiprotein signaling complexes. A yeast two-hybrid study using full length DLC2 as bait identified a number of interacting proteins that shared no singular functional class [23]. The SAM domain of DLC2, owing to its established role in protein-protein interactions, is an obvious choice for beginning a refined study of DLC2 partners.

As previously demonstrated by the homo-oligomeric forms of the Ste11 [24], Tel [25], Ephrin A4 [26] and Ephrin B2 [18] structures, SAM domains are capable of displaying a variety of protein interaction surfaces despite their relatively small size. Over the last five years, the repertoire of SAM domain/ligand interactions has been extended to include nucleic acids and lipids. Reinforcing the versatility of the fold, the surfaces employed by this emerging class of SAM domains are distinct from their protein-binding counterparts. For example, the Vts1 and Smaug SAM domains bind specific pentaloop hairpin RNAs at a shallow site that draws upon contributions from helices H1 and H5 [19]. The p73 SAM domain has been demonstrated to partially embed in both anionic and zwitterionic lipid membranes accompanied by predicted conformational changes in helices H1 and H3 [27].

Four isoforms of human DLC2 (DLC2α-δ) have been reported [21]. DLC2α and DLC2^β ^exhibit only minor amino terminal deletions before the SAM domain. DLC2^γ ^lacks the SAM domain while DLC2δ is a very short deletion (1–135) only possessing a SAM domain. At present, no information is available regarding cells overexpressing the DLCδ isoform. Does the overexpression of the SAM domain bind and suppress other signaling protein partners leading to a phenotypic change? Of note, only the DLC2^γ ^isoform could be stably expressed in HepG2 hepatoma cells suggesting the SAM domain may increase the suppressive strength of signals through Rho.

In yeast, the Ste50-SAM domain acts as a high-affinity beacon that recruits the Ste11 MAP kinase to Cdc42 GTPase-associated complexes involved in filamentous growth and the response to high osmolarity environment [28]. With its unique four-helix bundle fold and hydrophobic surface lined with aromatic residues, the DLC2-SAM domain may serve an analogous role by coupling receiver and effector proteins to inactive Rho- and Cdc42-associated complexes.

## Conclusion

The DLC2-SAM domain structure appears to be a hybrid of a SAM domain and an anti-parallel four-helix bundle. As a result, DLC2 may interact with a new class of biomolecular ligands, including peptides and lipids.

### Note Added in Proof

During revision of this manuscript, a structural study of the human DLC2-SAM domain was published [29]. Since coordinates of human DLC2 [PDB: 2H80] were unavailable, we could not perform a detailed comparison with the murine DLC2-SAM domain fragment presented in this study. In general terms, the number and position of the secondary structures of the human DLC2-SAM domain, as well as its topology, are in agreement with our murine DLC2-SAM data. Furthermore, we obtained a comparable correlation time indicating that both the human and murine DLC2-SAM domains occur in a predominantly monomeric form over a wide range of concentrations.

## Methods

### Cloning and Expression

Gene fragments encoding residues 1–137, 22–137, 50–120, and 50–137 of murine DLC2 were PCR amplified from the ATCC (Rockville, MD) cDNA clone 8437456 (GenBank: BC027830) and inserted into pET15b (Novagen). The expressed proteins have a 19 residue hexahistidine affinity tag and thrombin protease site appended to the amino terminus. Milligram quantities of isotopically labeled DLC2 SAM domain [50–137] were produced from a 1 L fermentation of *E. coli *BL21:DE3 in M9 minimal media supplemented with ^15^N-ammonium chloride and ^13^C-glucose as the sole nitrogen and carbon sources. The remaining fragments were isotopically labeled with ^15^N for screening purposes. All affinity tagged protein were purified using chelating nickel affinity chromatography and gel filtration chromatography (Sephadex S-100 16/60 column; GE Biosciences). Buffer conditions for all DLC SAM protein fragments were standardized to 20 mM sodium phosphate, pH 7.8, 175 mM NaCl, 0.05 % sodium azide, 5 mM dithiothreitol-d_10_.

### NMR spectroscopy and structure determination

Protein preparations of DLC SAM (50–137) for NMR structure determination were concentrated to 0.8 mM in the aforementioned standard buffer supplemented with 10% D_2_O. All NMR experiments were performed on a 600 MHz Varian NMRS instrument equipped with a room temperature, single axis, pulsed field gradient, triple axis probe. Standard Varian BioPack pulse sequences were employed. Backbone assignments were determined from 2D ^15^N-HSQC, 2D ^13^C-HSQC, 3D HNCACB, 3D CBCA(CO)NH, and 3D HNCO spectra. Side chain assignments were achieved from 3D H(CCO)NH, 3D C(CO)NH, and 3D HCCH-TOCSY spectra. Aromatic rings were assigned from 2D (HB)CB(CGCD)HD, 2D (HB)CB(CGCDCE)HE, and 3D HCCH-TOCSY spectra. NOE distance restraints were obtained from 3D ^15^N HSQC-NOESY (100 ms mixing time), 3D ^13^C HSQC-NOESY (100 ms) and 3D aromatic ^13^C HSQC-NOESY (80 ms) spectra. Amide residual dipolar couplings were obtained as J_NH _differences from 2D IPAP-HSQC spectra [30] of the original (isotropic) sample and an aligned sample containing 10 mg/mL Pf1 bacteriophage (Profos). Data were processed and interpreted using nmrPipe [31] and NMRView software [32]. No stereospecific assignments were made. NOE distance restraints were calibrated from 2.4–5.5 Å using CYANA 2.1 [33]. Hydrogen bond restraints (O-HN, 1.8–2.1 Å; O-N, 2.7–3.0 Å) were determined by assessing the initial ensemble for backbone O-HN distances < 2.4 Å and a bond angles < 25°. Backbone dihedral angles (φ/ψ) were predicted from chemical shift information using the PREDITOR method [34]. Initial ensembles of structures were calculated with CYANA 2.1 and further refined in explicit solvent [35] with XPLOR-NIH 2.17.0 [36].

### NMR dynamics

^15^N T_1 _relaxation spectra were acquired with delays of 10, 30, 50, 110, 210, 310 and 510 ms. ^15^N T_2 _relaxation spectra were acquired with delays of 10, 30, 50, 70, 90 and 110 ms. Amide heteronuclear NOE spectra were acquired with and without saturation and a total interscan delay of 5 s. Resonances were integrated and normalized with the nLinLS module of the NMRDraw software suite. Amide T_1 _and T_2 _relaxation times were calculated from least squares fitting to a monoexponential function. Heteronuclear NOE enhancements were calculated as a ratio between resonance intensities of the two spectra. A molecular rotational correlation time was calculated using a Mathematica notebook written by Dr. Pascal Mercier (Chenomx; Edmonton, AB) employing a subset of the relaxation data whose T_1_/T_2 _ratios were all within one standard deviation and whose heteronuclear NOE enhancements were > 0.65.

### Circular Dichroism Spectropolarimetry

Far UV spectra of DLC2 SAM domain fragments at a concentration of 50 μM in the standard buffer were acquired with a Jasco J-810 instrument. A rectangular cell with a 0.1 cm path length was used for all measurements. Spectra were recorded from 260 – 200 nm with a scan rate of 100 nm/min and a 1.0 nm bandwidth. A midpoint denaturation temperature (T_m_) was determined by heating samples from 20–80°C at 2°C/min and monitoring ellipticity at 220 nm, a wavelength that is diagnostic for α-helical content. Mean residue ellipticity at 222 nm for various DLC2-SAM protein fragments were predicted according to a previously described method [37].

### Bioinformatics

Ramachandran analysis of the ensemble was performed using PROCHECK-NMR [38]. Ensemble RMSDs were calculated with MOLMOL [39]. The EBI SSM server [14] was used to identify structurally similar proteins in the PDB and to perform 3D pairwise comparisons. Interhelical angles were calculated with INTERHLX [40]. Molecular graphics were produced with MOLMOL and PyMOL .

### Data Deposition

Coordinates and restraint lists were deposited in the Protein Data Bank under accession code 2JMT. Chemical shifts were deposited in the BioMagResBank (BMRB) under accession number 15060.

## Authors' contributions

JJK cloned, expressed and purified the DLC2-SAM domain fragments. LWD acquired the NMR data, JJK contributed backbone chemical shift assignments and performed the CD spectroscopy. LWD solved the structure, made the database depositions. All authors approved the manuscript.
